# Supplementary material for: Microbiome convergence enables siderophore-secreting-rhizobacteria to improve iron nutrition and yield of peanut intercropped with maize
Source: Nat Commun. 2024 Jan 29;15:839. doi: 10.1038/s41467-024-45207-0 (PMC10825131; doi:10.1038/s41467-024-45207-0)
Supplement: Supplementary file 1 — Supplementary Information [file 41467_2024_45207_MOESM1_ESM.pdf]

Microbiome convergence enables siderophore-secreting-rhizobacteria to improve iron nutrition and yield of peanut intercropped with maize

Nanqi Wang[1,a], Tianqi Wang[1,a], Yu Chen[2,a,], Ming Wang[3,*], Qiaofang Lu[1], Kunguang Wang[1], Zhechao Dou[1], Zhiguang Chi[1], Wei Qiu[1], Jing Dai[1], Lei Niu[1], Jianyu Cui[1], Zhong Wei[4,*], Fusuo Zhang[1], Rolf Kümmerli[5] and Yuanmei Zuo[1,*]

[1]College of Resources and Environmental Sciences, State Key Laboratory of Nutrient Use and Management, China Agricultural University, 100193, Beijing, China

[2]Jiangsu Key Laboratory for the Research and Utilization of Plant Resources, Jiangsu Province Engineering Research Center of Eco-cultivation and High-value Utilization of Chinese Medicinal Materials, Institute of Botany, Jiangsu Province and Chinese Academy of Sciences, 210014, Nanjing, Jiangsu, China

[3]Department of Plant Pathology, The Key Laboratory of Plant Immunity, Nanjing Agricultural University, Nanjing, 210095, China

[4]College of Resources and Environmental Science Nanjing Agricultural University, Key lab of organic-based fertilizers of China and Jiangsu provincial key lab for solid organic waste utilization, Nanjing Agricultural University, Nanjing 210095, China

[5]Department of Quantitative Biomedicine, University of Zurich, Zurich, Switzerland

[a]These authors contribute equally to this work.

*Corresponding author

Correspondence to: Zhong Wei (weizhong@njau.edu.cn), Ming Wang (mwang@njau.edu.cn), and Yuanmei Zuo (zuoym@cau.edu.cn)

This PDF contains Supplementary Figures 1-25 and Supplementary Tables 1-7.

Supplementary Figures

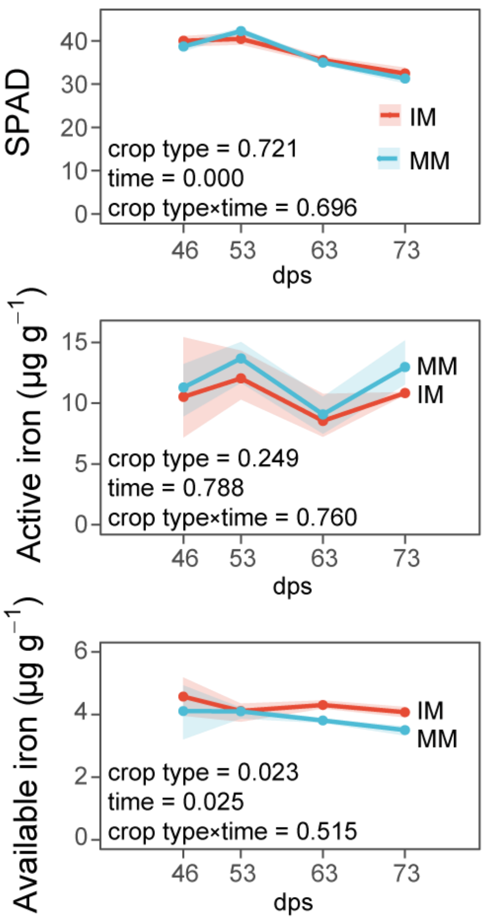

Supplementary Fig. 1 | Intercropping has no effect on maize iron nutrition. Soil Plant Analysis Development (SPAD) values, active Fe in leaves and available Fe in the rhizosphere of monocropping maize and intercropping maize. Points and shaded areas show mean values and 95% confidence interval from four independent biological replicates, respectively. Crop type indicates monocropping or intercropping. Statistical analyses were performed using two-way ANOVAs because data meet the assumptions of parametric tests. For all parameters, the number of biologically independent samples is four. Two-sided tests were used for alternative hypothesis testing. Source data are provided.

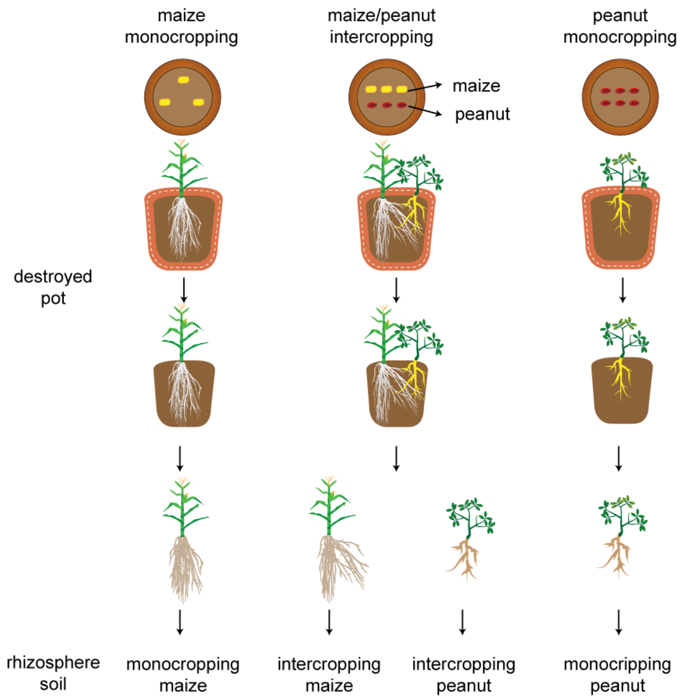

Supplementary Fig. 2 | Sampling design and process for intercropping of greenhouse experiment. Three cropping treatments were set up in 8 kg Fe-limiting soil. Six peanut plants or three maize plants were grown in a pot for the monocropping peanut and monocropping maize. Three peanut plants with three maize plants were grown in a pot for the intercropping treatments. The pot was destroyed to acquire complete soil. The roots of intercropping maize and intercropping peanut were carefully separated from each other when shaken. The rhizosphere soil was obtained by gently brushing the soil on the root surface with a sterile brush.

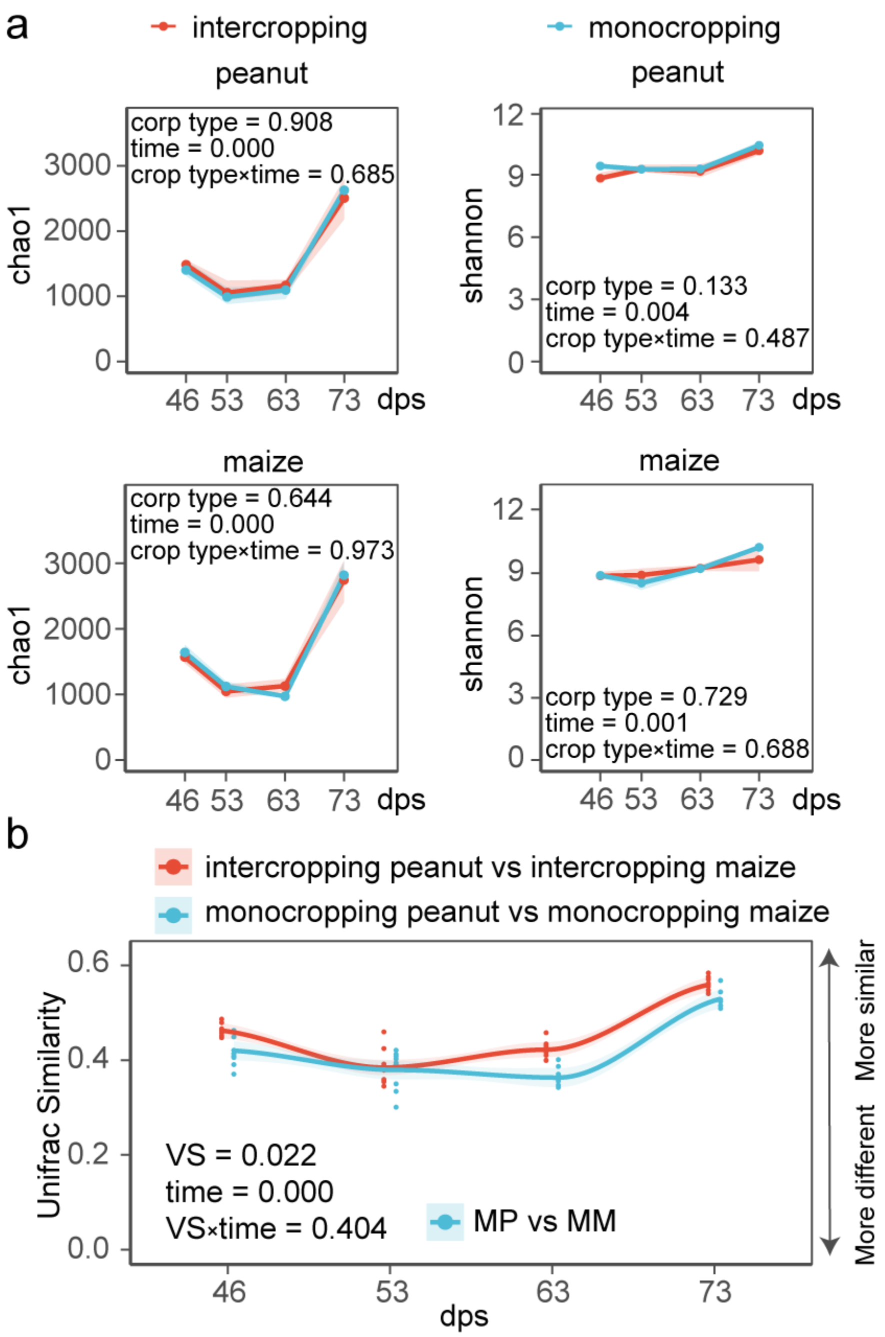

Supplementary Fig. 3 | Intercropping did not affect α diversity but increased the β diversity similarity of rhizobacteria. a, α diversity of rhizosphere of monocropping peanut, intercropping peanut, monocropping maize, and intercropping maize at 46, 53, 63 and 73 days post sowing (dps). Red and blue indicate intercropping and monocropping samples, respectively. Circles indicate mean value. α diversity refers to the diversity within a particular ecosystem (i.e. within habitats or sites), and is usually

expressed by the number, richness and evenness of species in that ecosystem. **b,** taxonomic similarity (β diversity similarity) based on UniFrac distance of monocropping peanut *vs.* monocropping maize (blue) and intercropping peanut *vs.* intercropping maize (red). β diversity refers to a comparison of diversity between ecosystems (i.e., between habitats or sites), usually measured as the amount number of species that differ change between the ecosystems. Dots represent individual values at different dps."VS" indicates the monocropping vs. intercropping. For (**a**) and (**b**). Two-way tests were performed by the Scheirer-Ray-Hare test, and *p* values are shown. Each curve is generated using a LOESS smoothing procedure. Shaded areas are 95% confidence intervals. For all parameters, the number of biologically independent samples is three. Two-sided tests were used for alternative hypothesis testing. Source data are provided.

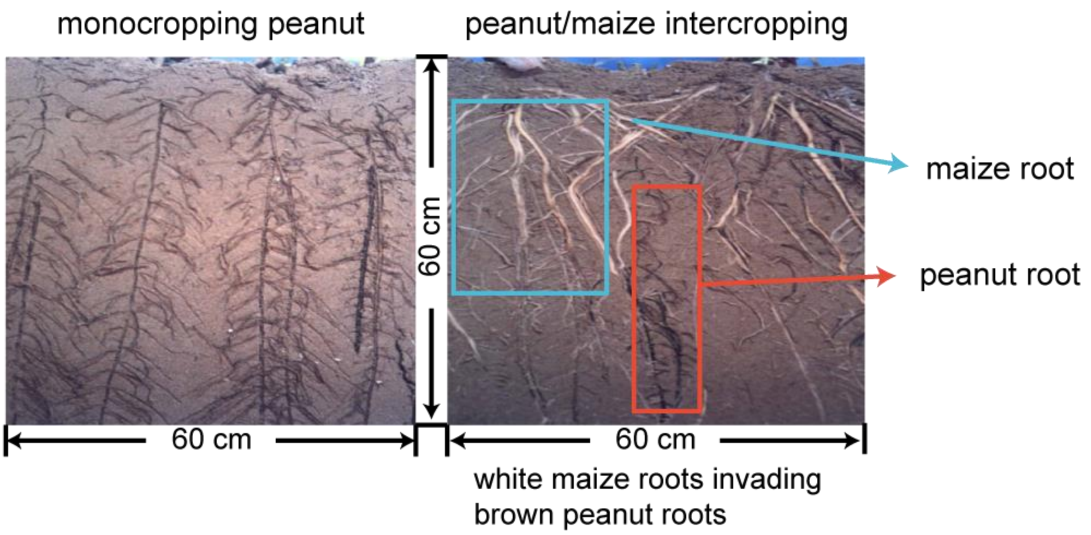

Supplementary Fig. 4 | The white maize root invaded into the brown peanut rhizosphere root network across a distance of 60 cm. The physical interaction between fine roots can lead to the transfer of the functional microbiome from one species to the other. The width and height of rhizobox was 60 cm, which represents the distance across which root interactions between maize and peanut physically occur in the field.

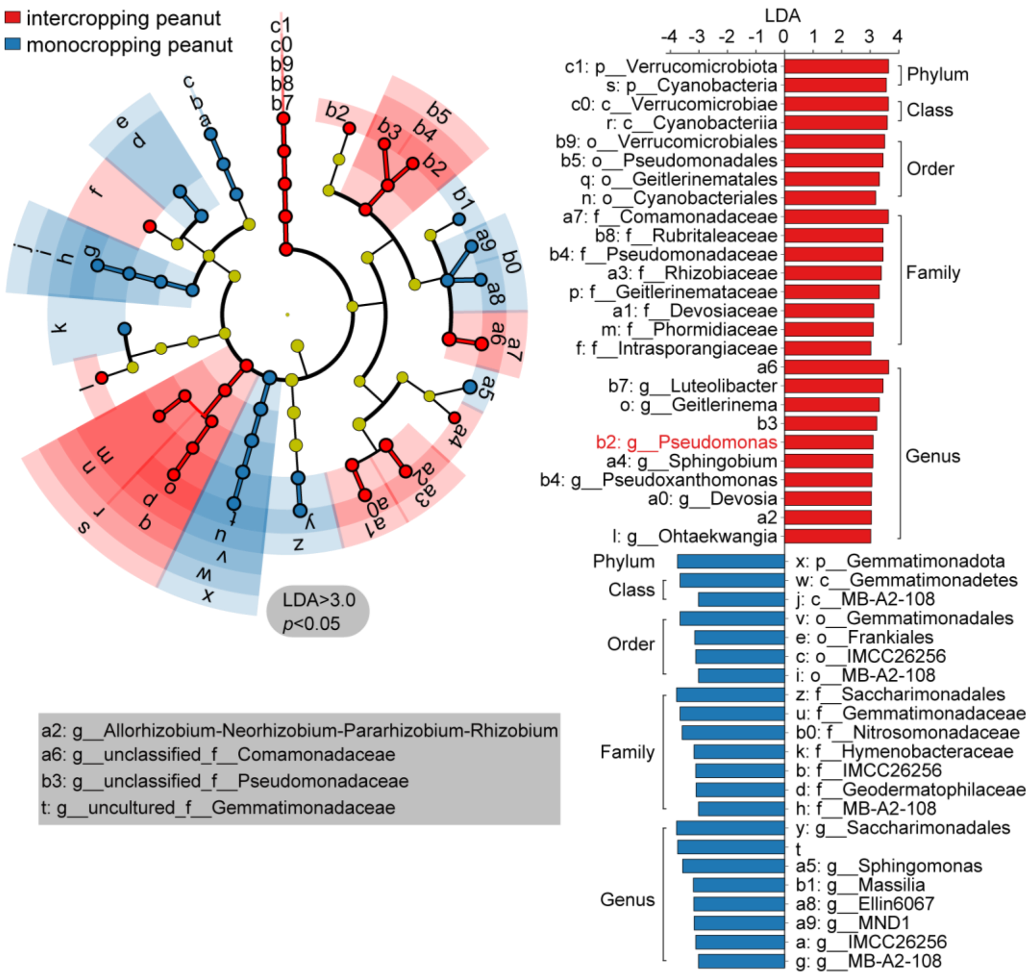

Supplementary Fig. 5 | Bacteria biomarkers discriminating intercropping peanut and monocropping peanut identified by LefSe. The threshold values for LefSe were LDA > 3.0 and $p < 0.05$. Enriched taxa in intercropping peanut or monocropping peanut bacteria are shown in red or blue. A cladogram indicates the phylogenetic relationship among biomarker taxa, and the levels represent, from inner to outer rings, phylum, class, order, family, and genus. Bar chart shows the LDA scores of bacterial taxa identified by LefSe. The number of biologically independent samples for LefSe analysis is three. Two-sided tests were used for alternative hypothesis testing. Source data are provided.

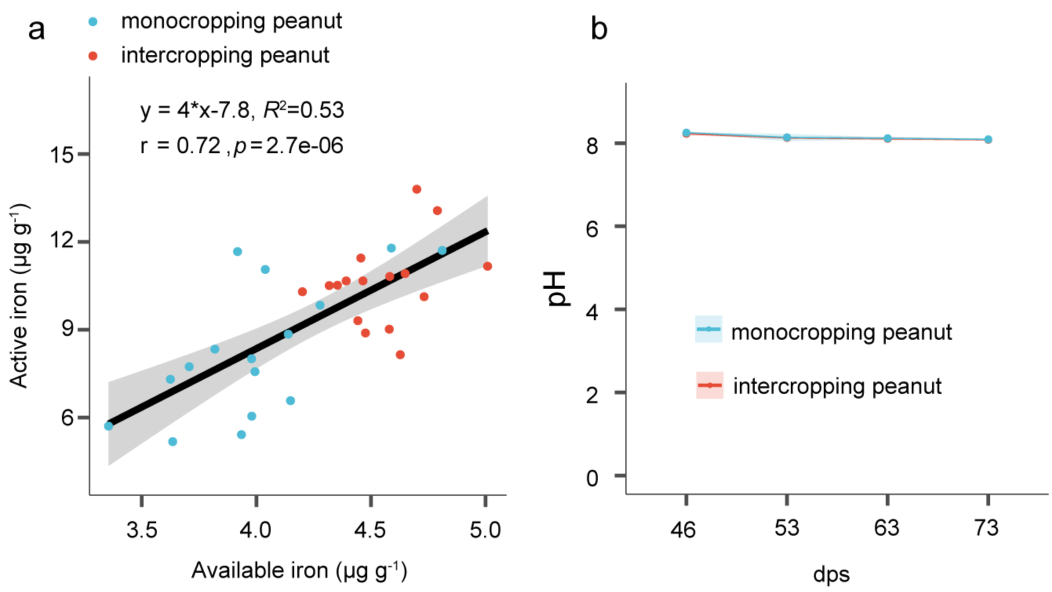

Supplementary Fig. 6 | Active iron concentration in young leaves of peanut was positively correlated with available iron concentration in the peanut rhizosphere. **a,** correlation analysis between available iron and active iron. Dots represent individual values of monocropping peanut (blue) and intercropping peanut (red) samples. Regression line is determined based on a simple linear regression analysis. Shaded areas are 95% confidence intervals. The Pearson's correlation coefficient, regression equation, coefficient of determination, and *p*-value are shown, based on 32 biologically independent samples. Two-sided tests were used for alternative hypothesis testing. **b,** soil pH remained stable over time and there is no difference in the rhizosphere pH value between monocropping peanut and intercropping peanut. Points and shaded areas show mean values and SD from four independent biological replicates, respectively. Source data are provided.

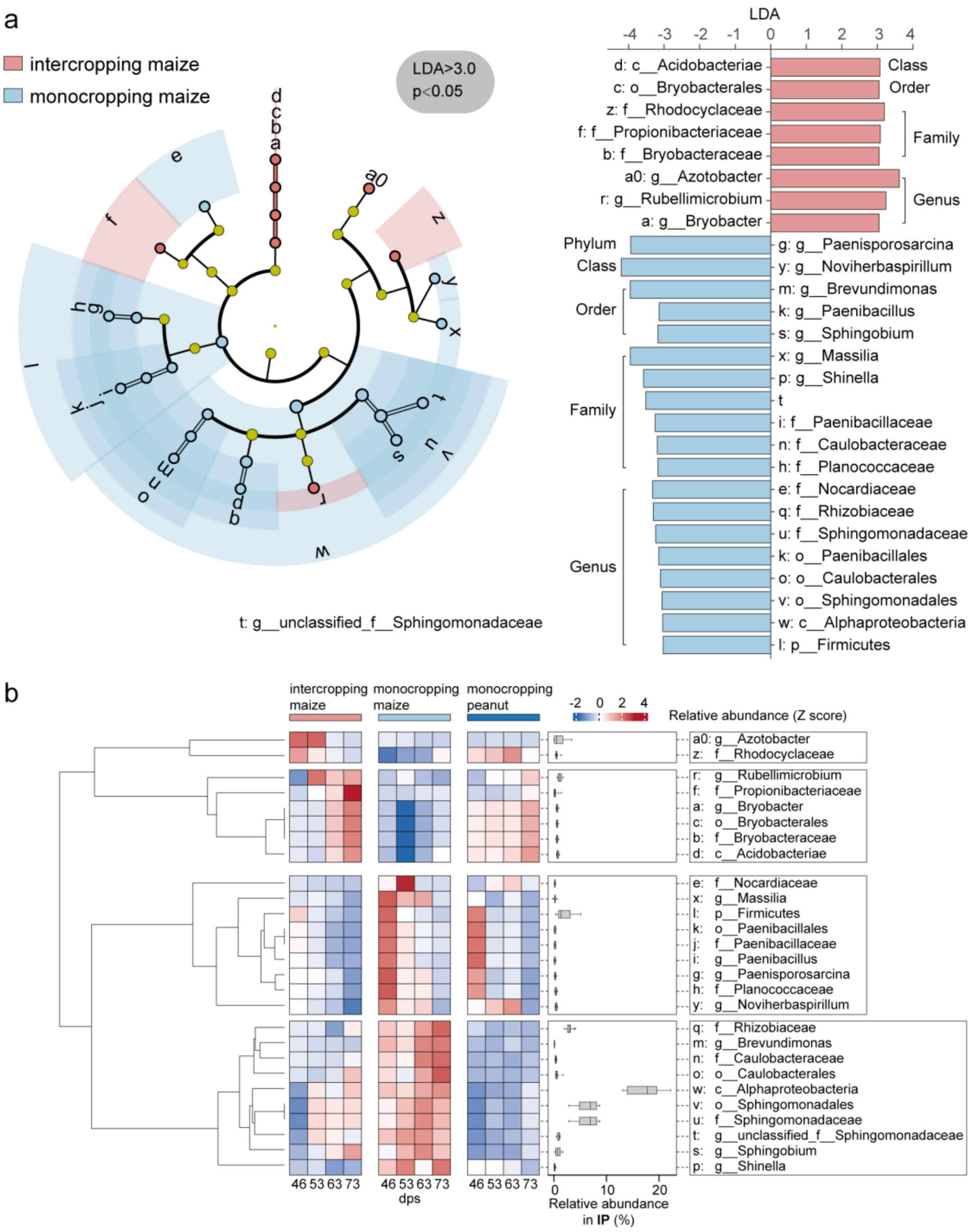

Supplementary Fig. 7 | Bacteria biomarkers discriminating intercropping maize and monocropping maize identified by LefSe. a, Enriched taxa in intercropping maize or monocropping maize bacteria are shown in red or blue. The threshold values for LefSe were LDA > 3.0 and p < 0.05. A cladogram indicates the phylogenetic relationship among biomarker taxa, and the levels represent, from inner to outer rings, phylum, class, order, family, and genus. Bar char shows the LDA scores of bacterial

taxa identified by LefSe.**b,** Heatmap of relative abundance of keystone genus in intercropping maize, monocropping maize and monocropping peanut. Relative abundance is normalized by a z-score. The colour of the cell indicates the z-score (red, high z-score; blue, low z-score). For all parameters, the numbers of biologically independent samples are three. Two-sided tests were used for alternative hypothesis testing. Source data are provided.

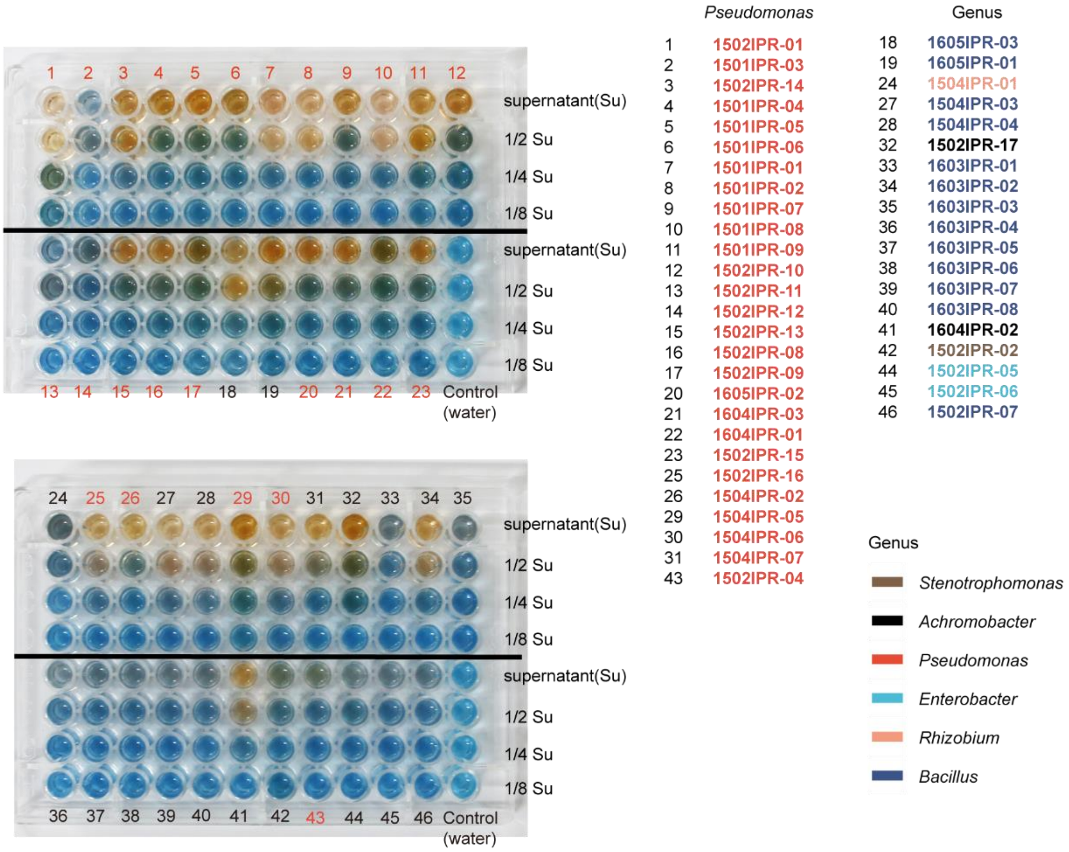

Supplementary Fig. 8 | The CAS assay with gradient dilution (stock, 1/2, 1/4 and 1/8) of supernatant cultured in Fe-limited conditions. The gradual colour change from blue to orange is proportional to the amount of siderophore in the supernatant. The control indicates the reaction of water in the CAS assay. The colour of the strain represents the genus the strain belongs to, based on the full-length 16S rRNA gene sequence. The CAS assays were repeated three times yielding similar results.

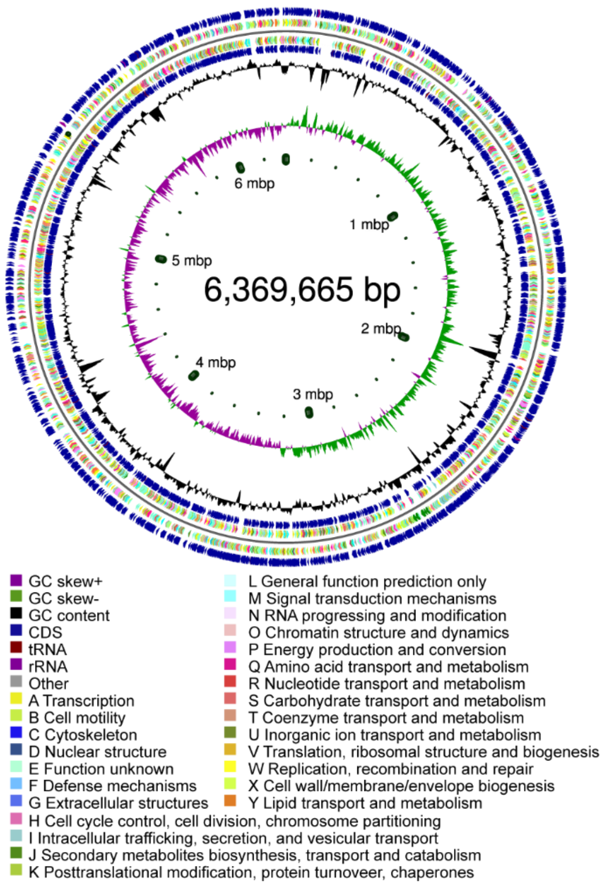

Supplementary Fig. 9 | The circularized genome map of *Pseudomonas* sp. 1502IPR-01. The outermost and second circles of all replicons indicate genes in forwarding orientations, and the third and fourth circles of all replicons indicate genes in reverse orientations. The second and third circles are colour-coded by their role categories. The fifth circle shows G+C content in black, and the sixth circle shows the G+C skew in green (+) and purple (−). The scale is shown in the innermost circle.

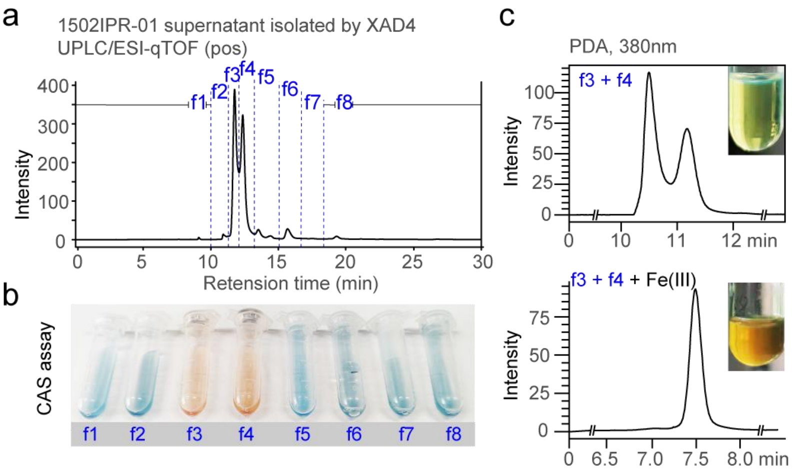

Supplementary Fig. 10 | Siderophore isolation of *Pseudomonas* sp. 1502IPR-01. a, UPLC/ESI-qTOF results (380 nm) of purified fraction of supernatant of *Pseudomonas* sp. 1502IPR-01 cultured in Fe-limiting conditions by Amberlite XAD4 adsorbent resin twice. **b,** CAS assay result of eight fractions of purified fraction isolated by preparative chromatogram. f3 and f4 fractions turned red, indicating that these two fractions contained siderophore. **c,** The colour and the photo-diode array detector (PDA) chromatogram (380 nm) of f3 and f4 fractions and the complex of f3+f4+Fe(III). Fractions f3 and f4 could not be separated by preparative chromatography but they are indeed the same substance due to the following reasons: 1) they yielded the same siderophore activity in the CAS assay; 2) any fine fractions from fraction f3 to f4 obtained by preparative chromatography have same chromatogram map and tandem mass spectrum also indicates that the substance in f3 to f4 have the same molecular weight; and 3) f3 and f4 merged into a single peak in HPLC when chelated with Fe(III), we therefore concluded that fractions f3 and f4 contain the same molecule, which was later confirmed by NMR.

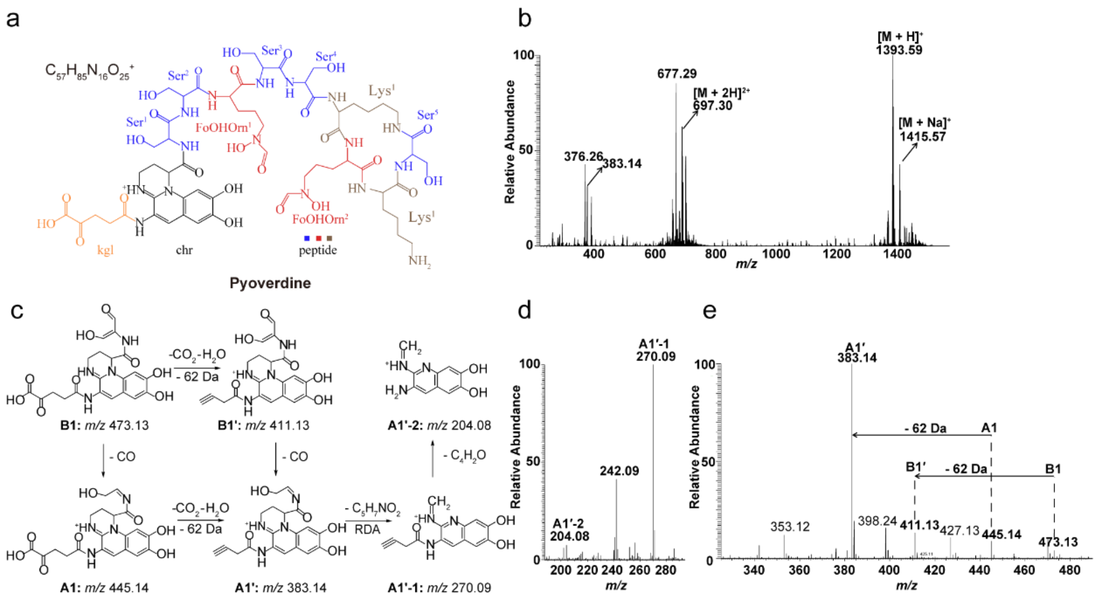

Supplementary Fig. 11 | a, Structure of pyoverdine, the putative structure of siderophore of *Pseudomonas* sp. 1502IPR-01. The structure consists of three parts: (1) a dihydroquinoline-type chromophore: (1S)-5-amino-2,-3-dihydro-8,9-dihydroxy-1H-pyrimido-[1,2-a]quinolone-1-carboxylic acid (Chr); (2) a peptide comprised of 9 amino acids: Ser-Ser-FoOHOrn-Ser-Ser-(Lys-FoOHOrn-Lys-Ser)) (Ser: serine; FoOHOrn: 5-N-formyl-5-N-hydroxyornithine; Lys: lysine); and (3) a side-chain (α-ketoglutaric acid, kgl) bound to the nitrogen atom at position C-3 of the chromophore. The pyoverdine of *Pseudomonas* sp. 1502IPR-01 is identical to the previously described pyoverdine secreted by *Pseudomonas* spp. CFML 95-275[1]. Based on the elucidated pyoverdine structure[2], we speculated that the two hydroxyls of the chromophore, together with the hydroxyls and carbonyl of the two FoOHOrn in the peptide backbone form the binding site for Fe(III), generating the three bidentate ligands forming the octahedral complex with Fe(III). **b,** $[M + H]^+$ and $[M + 2H]^{2+}$ ions of pyoverdine at high energy collision-induced dissociation (HCD) 15 ev. **c**, Proposed structure-fragmentation pathways of the chromophore of pyoverdine. **d,** MS fragments of pyoverdine at HCD 45 ev. **e,** MS

fragments of pyoverdine at HCD 20 ev.

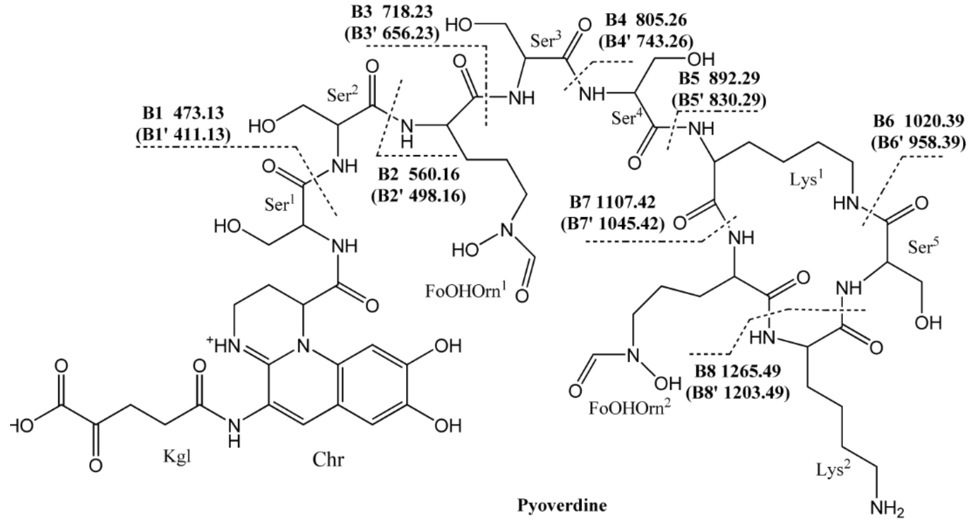

Supplementary Fig. 12 | Ions observed after collision activation of $[M + H]^+$ and $[M - H_2O - CO_2 + H]^+$ ions of pyoverdine.

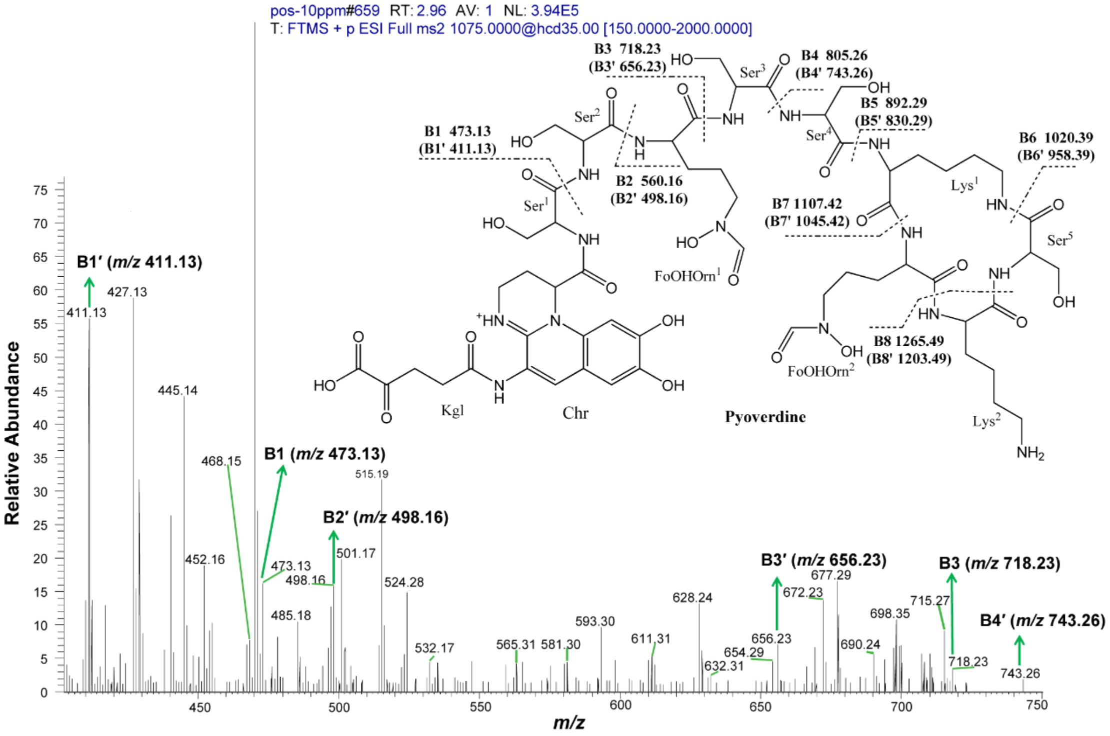

Supplementary Fig. 13 | Ions observed after collision activation of $[M + H]^+$ and $[M - H_2O - CO_2 + H]^+$ ions of pyoverdine.

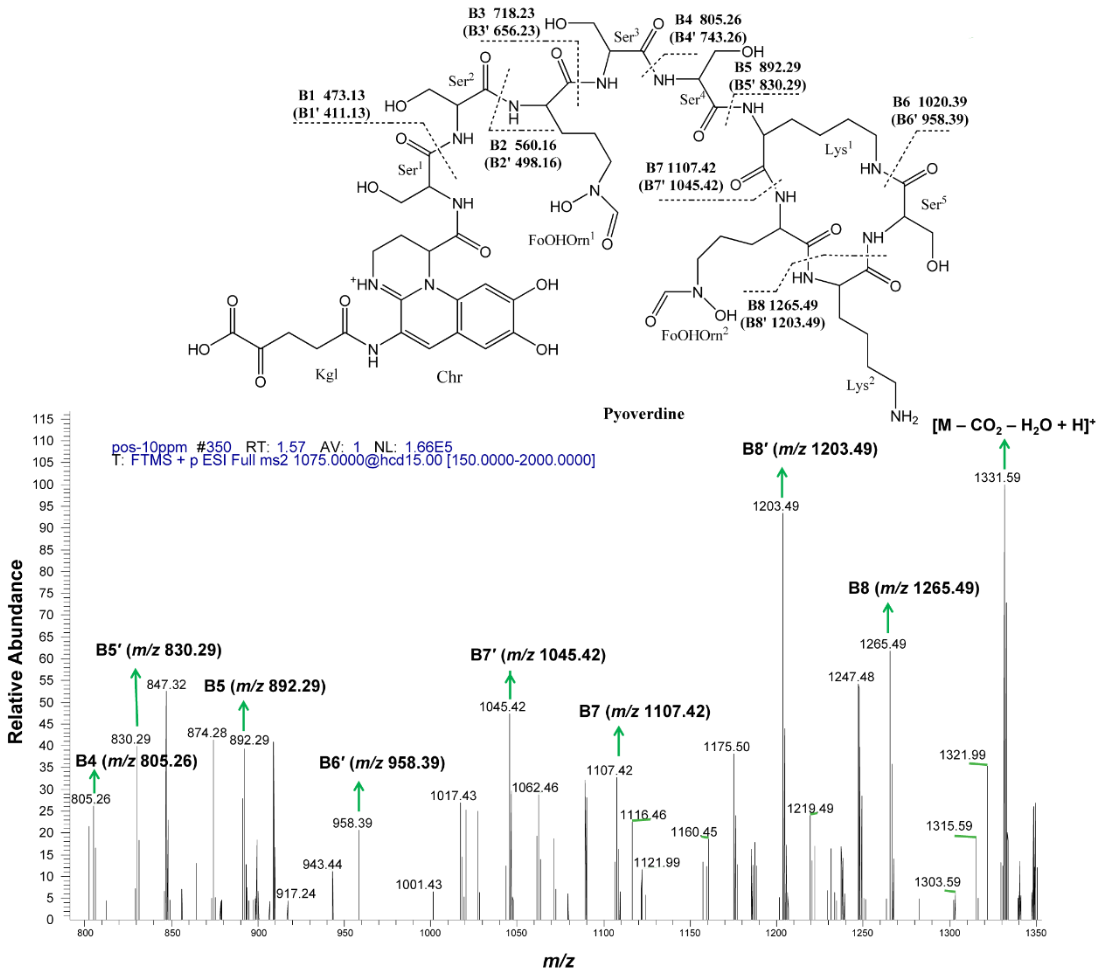

145 **Supplementary Fig. 14 |** MS fragments of pyoverdine at HCD 15 ev.

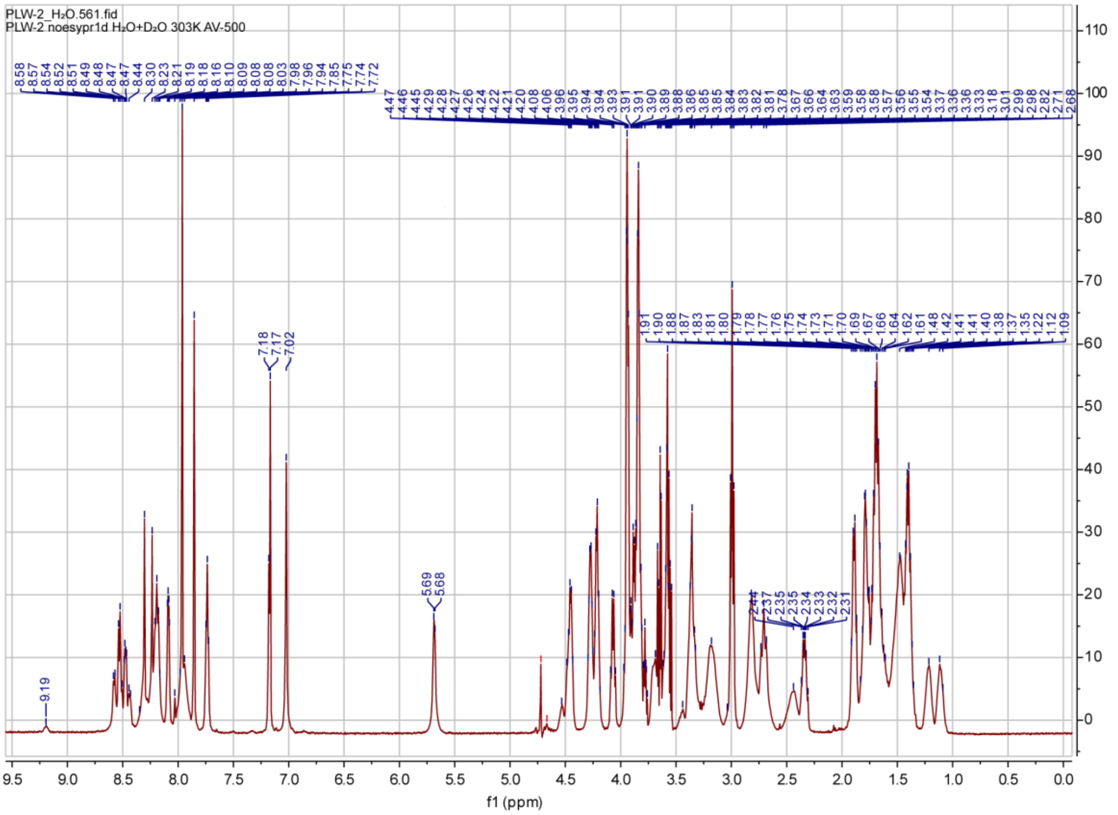

Supplementary Fig. 15 | ^{1}H-NMR spectrum (500 MHz, H_2O/D_2O, 9:1) of pyoverdine.

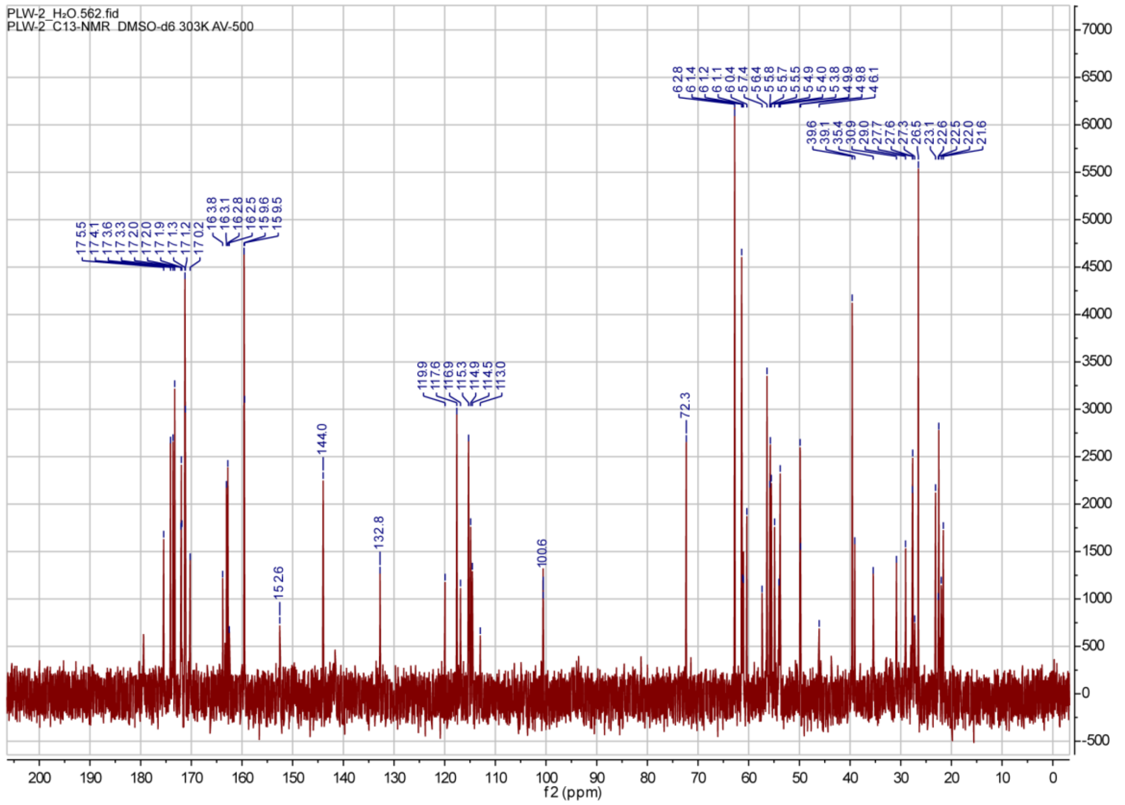

Supplementary Fig. 16 | ^{13}C-NMR spectrum (125 MHz, H_2O/D_2O, 9:1) of pyoverdine.

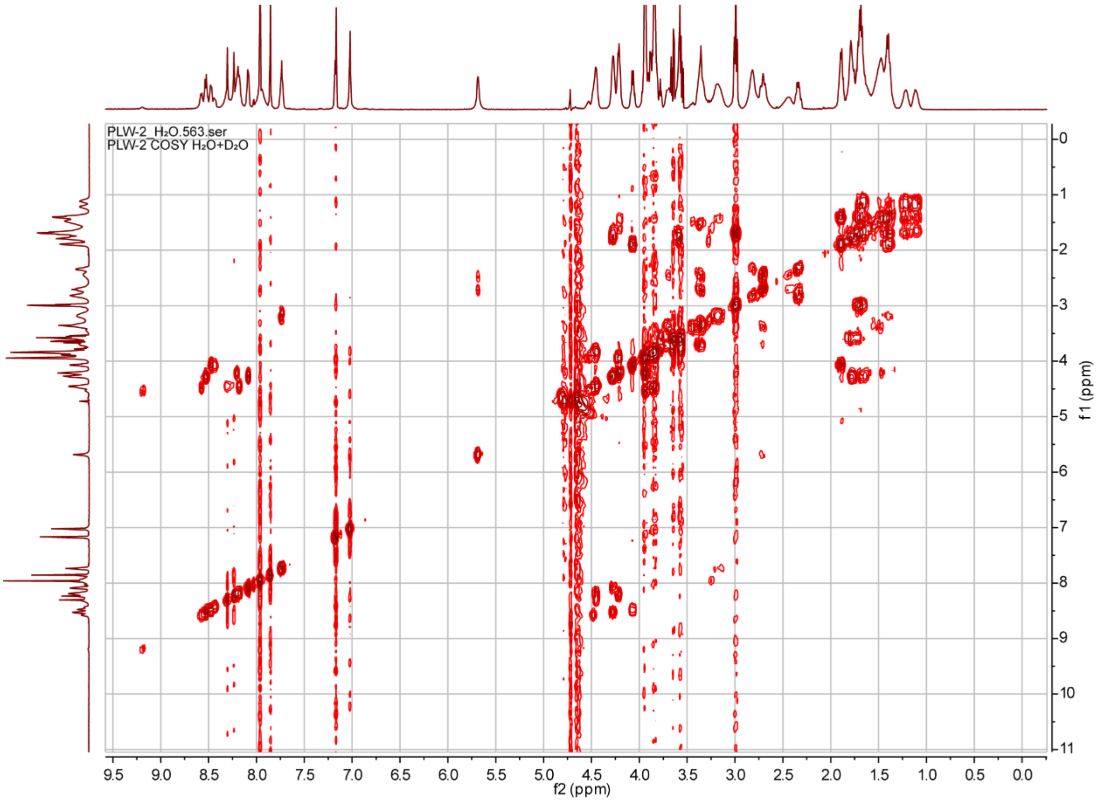

Supplementary Fig. 17 | ^{1}H -^{1}H COSY spectrum (500 MHz, H_2O/D_2O, 9:1) of pyoverdine.

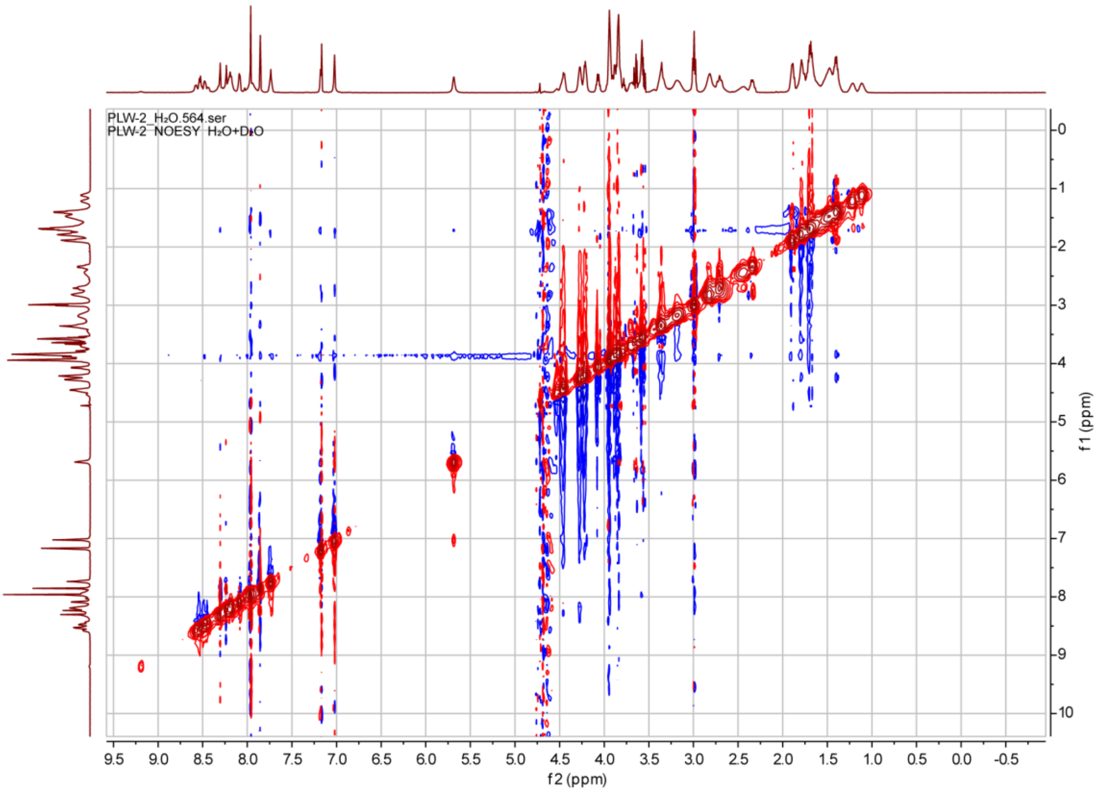

Supplementary Fig. 18 |. NOESY spectrum (500 MHz, H_2O/D_2O, 9:1) of pyoverdine.

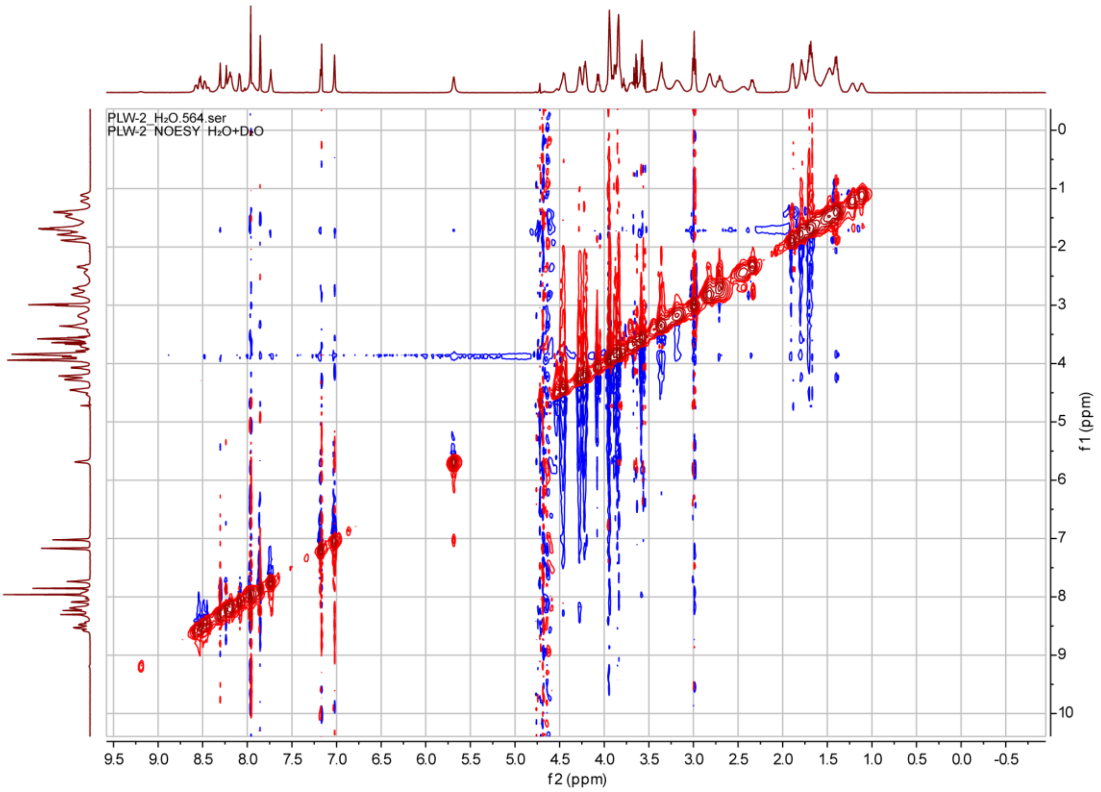

Supplementary Fig. 19 | HSQC spectrum (500 MHz, H_2O/D_2O, 9:1) of pyoverdine.

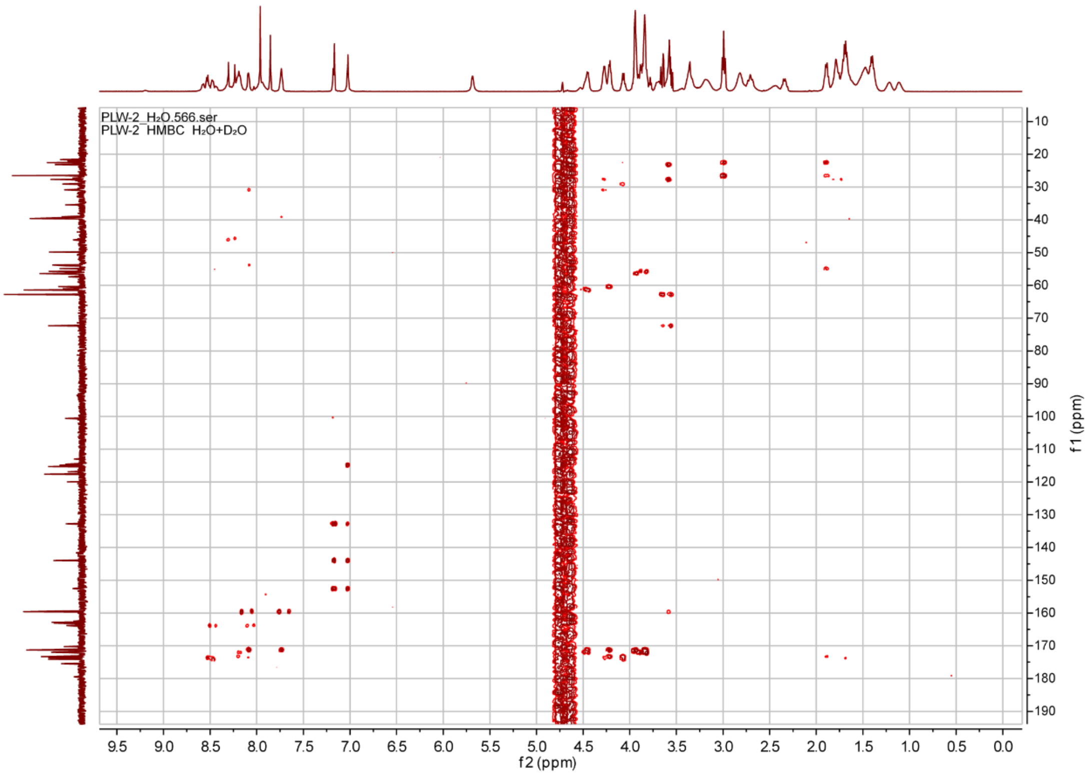

Supplementary Fig. 20 | HMBC spectrum (500 MHz, H_2O/D_2O, 9:1) of pyoverdine.

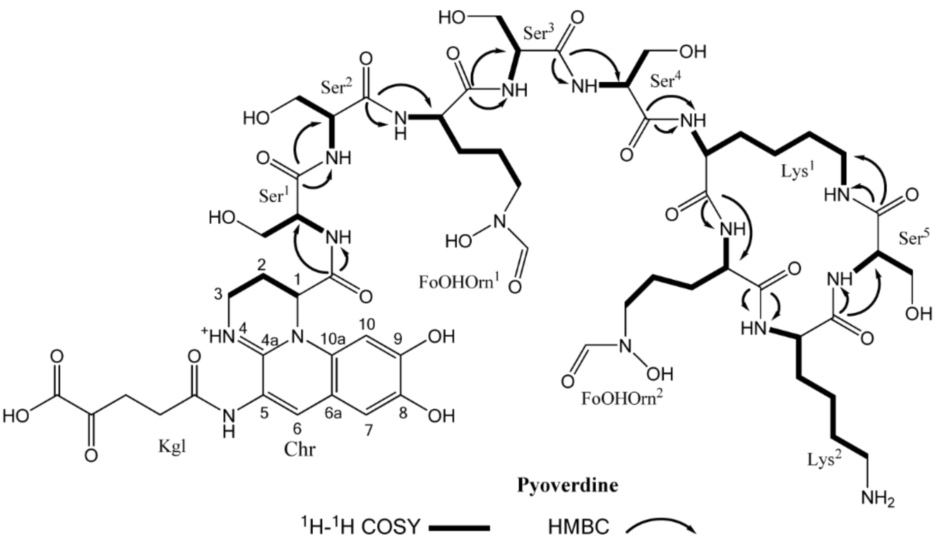

Supplementary Fig. 21 | Key ^{1}H–^{1}H COSY (black thick lines) and key HMBC correlations (black arrows) of pyoverdine.

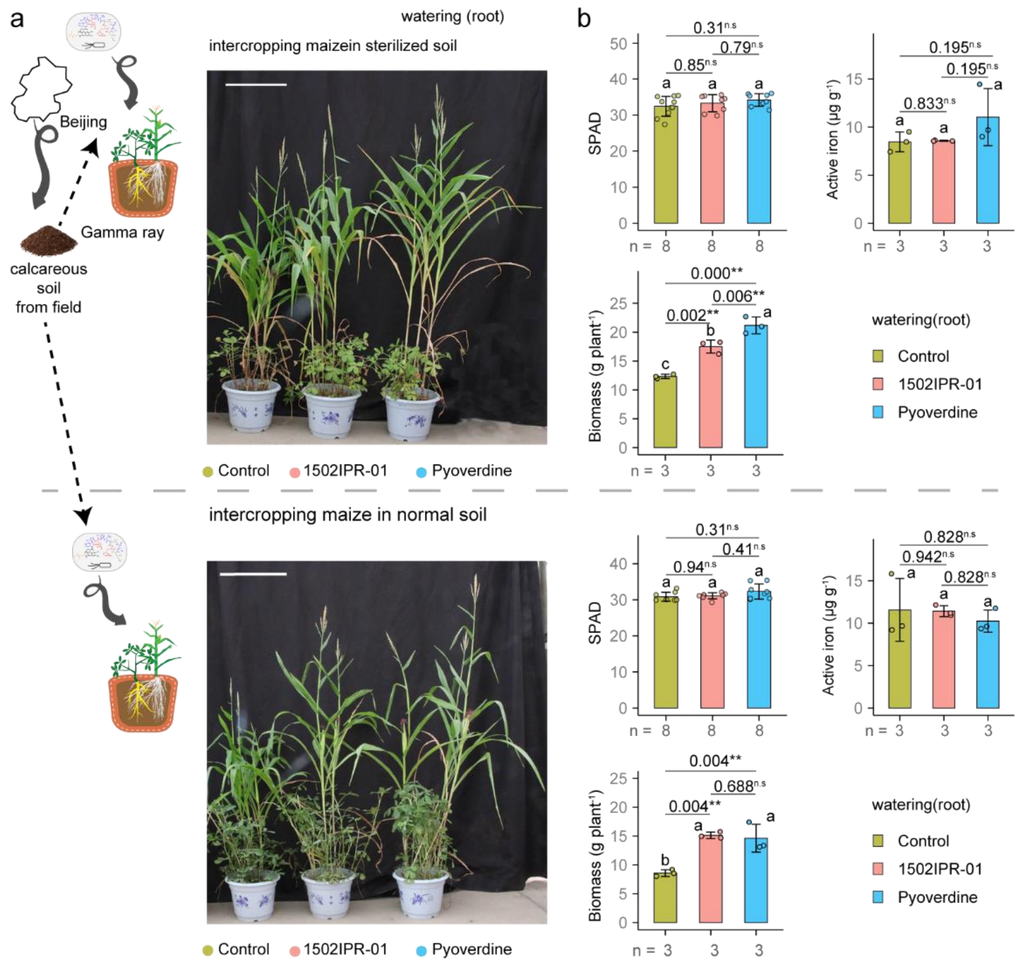

Supplementary Fig. 22 | The *Pseudomonas* sp. 1502IPR-01 isolate and pyoverdine improve biomass but not iron nutrition of maize intercropped with peanut. a, phenotype of maize intercropped with peanut. **b,** Soil Plant Analysis Development (SPAD) values, active iron concentration of young leaves, and biomass of intercropped maize. For (**b**), the number of independent biological replicates is indicated on the x-axis, and bars and error bars represent mean ± SD and dots represent individual values. ANOVA with LSD post-hoc test was used when data followed the normal distribution and had homogenous variances. For skewed data sets or data with heterogeneous variances, the BoxCox transformation algorithm was applied. When the data still did not meet the assumptions of parametric tests, Kruskal-Wallis test with Dunnett T3 test

was used. Different letters indicate significant differences between groups. Multiple testing corrections were performed by the BH algorithm. For all parameters, n represents the number of biologically independent samples. Two-sided tests were used for alternative hypothesis testing. Source data are provided..

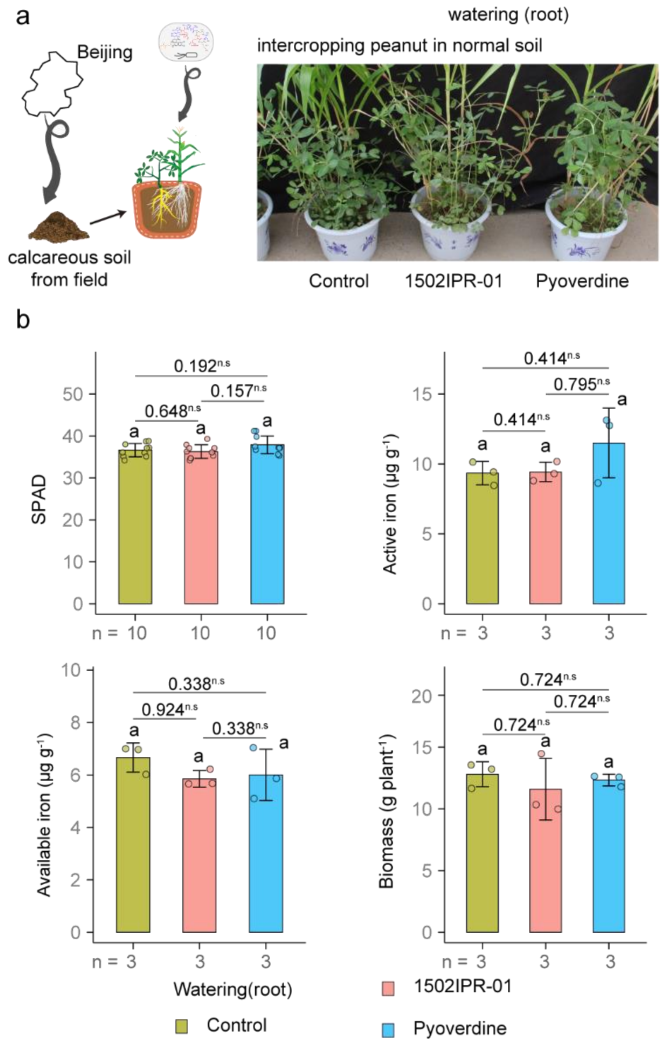

Supplementary Fig. 23 | The *Pseudomonas* sp. 1502IPR-01 isolate and pyoverdine had no effect on intercropping peanut iron nutrition in greenhouse conditions in normal non-sterile soil. a, Phenotype of soil application of *Pseudomonas* sp. 1502IPR-01, unchelated pyoverdine and control of intercropping peanut in normal soil. **b,** The Soil Plant Analysis Development (SPAD) values and active iron in young leaves and available iron in the rhizosphere. The number of independent biological replicates is indicated on the x-axis and bars ± error bars represent mean ± SD and dots represent individual values. ANOVA with LSD post-hoc test was used when data followed the normal distribution and had homogenous variances. For skewed data sets or data with

heterogeneous variances, the BoxCox transformation algorithm was applied. When the data still did not meet the assumptions of parametric tests, Kruskal-Wallis test with Dunnett T3 test was used. Different letters indicate significant differences between groups. Multiple testing corrections were performed by the BH algorithm. For all parameters, n represents the number of biologically independent samples. Two-sided tests were used for alternative hypothesis testing. Source data are provided.

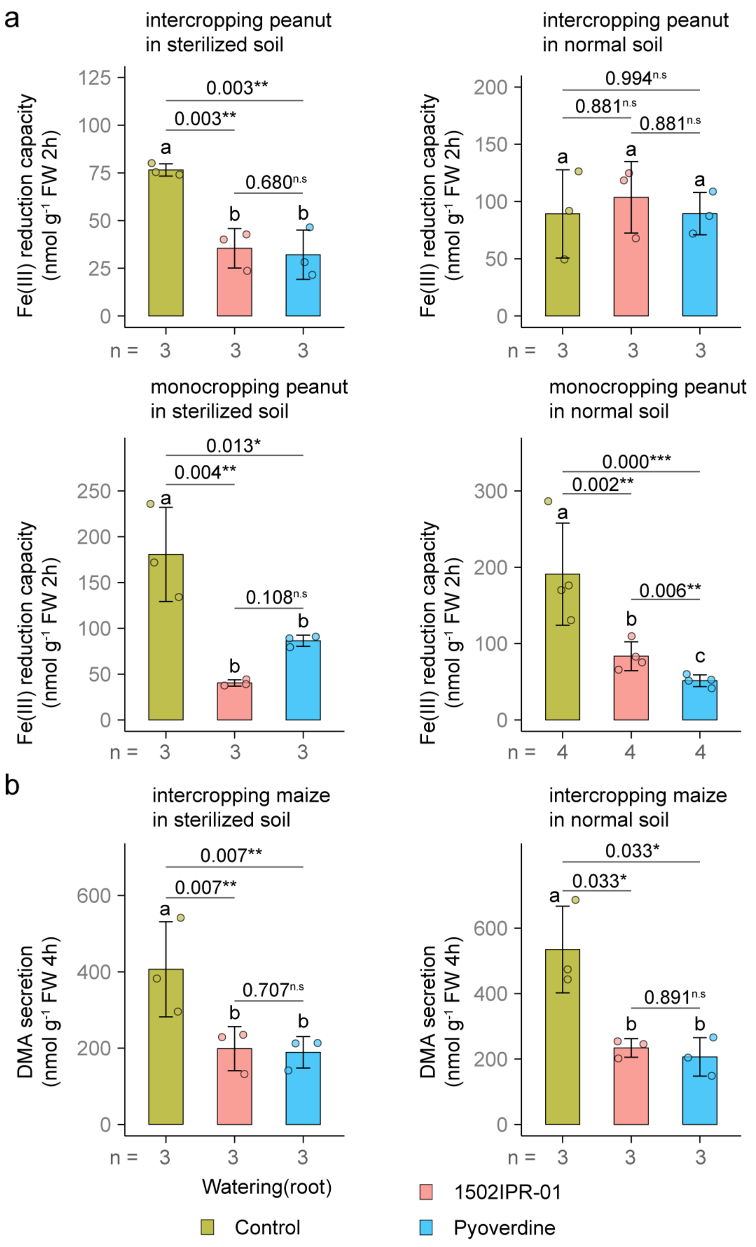

Supplementary Fig. 24 | The *Pseudomonas* sp. 1502IPR-01 isolate and pyoverdine decrease the key iron-deficiency responses of peanut and maize plants. a, Fe(III) reduction capacity of peanut. **b**, DMA (2'-deoxy mugineic acid, the phytosiderphores of maize) secretion of intercropped maize. The number of independent biological replicates is indicated on the x-axis and bar ± error bars represent mean ± SD and dots represent individual values. ANOVA with LSD post-hoc test was used when data followed the normal distribution and had

homogenous variances. For skewed data sets or data with heterogeneous variances, the BoxCox transformation algorithm was applied. When the data still did not meet the assumptions of parametric tests, Kruskal-Wallis test with Dunnett T3 test was used. Different letters indicate significant differences between groups. Multiple testing corrections were performed by the BH algorithm. For all parameters, n represents the number of biologically independent samples. Two-sided tests were used for alternative hypothesis testing. Source data are provided.

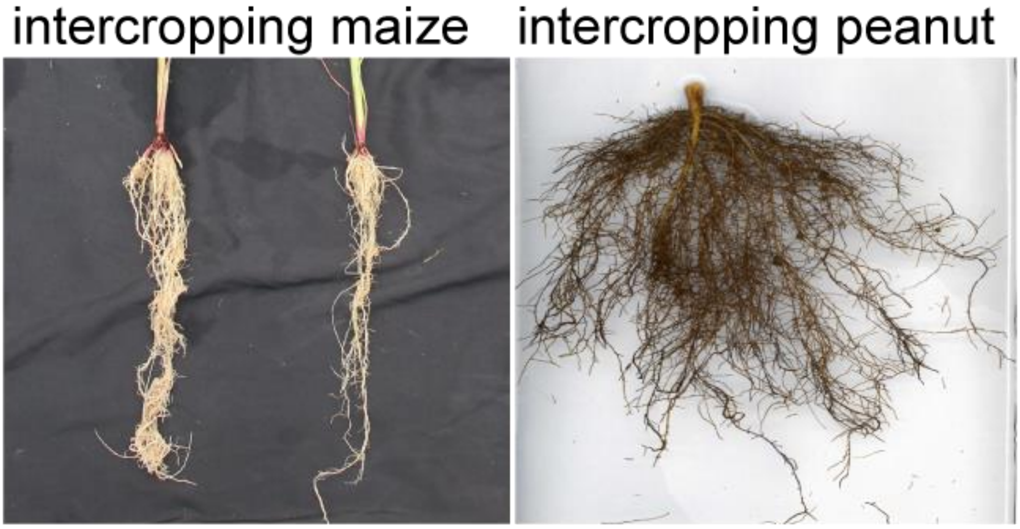

Supplementary Fig. 25 | The roots of maize (white) and peanut (dark brown). The distinct differences in colour and morphology allowed us to completely and reliably separate the roots of the two species and to ensure that the contamination risk was minimal.

Supplementary Tables

Supplementary Table 1 | The identity of ASV 487 and 338F-806R fraction of 16S rDNA of *Pseudomonas* strains isolated from intercropping peanut rhizosphere

Strain name	Isolated stages (days post sowing)	Length	Identity (%)	Gaps (%)	Scores
1502IPR-10	53	429	97.7	2.1	2428
1502IPR-13	53	429	97.7	2.1	2428
1502IPR-11	53	429	99.8	0	2480
1501IPR-01	46	429	100	0	2487
1501IPR-05	46	429	100	0	2487
1501IPR-04	46	429	100	0	2487
1501IPR-06	46	429	100	0	2487
1502IPR-08	53	429	98.8	0	2458
1502IPR-16	53	430	97.9	0.5	2056
1502IPR-01	53	429	97.7	0	2424
1502IPR-14	53	429	97.7	0	2424
1604IPR-01	73	430	97.9	0.5	2057
1502IPR-09	53	430	95.3	0.2	2347
1604IPR-03	73	429	97.4	0	2419
1502IPR-15	53	429	97.9	0	2420
1605IPR-02	73	429	97.7	0	2410
1504IPR-06	73	429	97.9	0	2420
1502IPR-12	53	429	97.2	0	2395
1502IPR-04	53	429	95.1	0	2342
1501IPR-07	46	429	95.1	0	2342
1501IPR-09	46	429	95.1	0	2342
1501IPR-02	46	429	95.1	0	2342
1501IPR-08	46	429	94.4	0	1929
1501IPR-03	46	429	94.4	0	1929
1504IPR-07	73	429	93.5	0	2313
1504IPR-02	73	429	92.8	0	2296
1504IPR-05	73	429	93.9	0	2324

Supplementary Table 2 | Cluster of pyoverdine biosynthesis and secretion genes in *Pseudomonas aeruginosa* PAO1 and *Pseudomonas* sp. 1502IPR-01

P.aeruginosa PAO1 Locus Tag	name	Description	PAO1 position	PAO1 sequence length	1502IPR-01 position	1502IPR-01 sequence length	Identiy of DNA sequence (%)
PA2385	pvdQ	acyl-homoserine lactone acylase PvdQ	c2638805-2636517	2288	c3344855-3347140	2285	65.7
PA2386	pvdA	L-ornithine N5-oxygenase	c4119969-4118641	1328	c4118632-4119969	1337	78.4
PA2387	fpvI	extracytoplasmic-function sigma-70 factor	c2640871-2640392	479	c4120090-4120569	479	73.3
PA2389	pvdR	Efflux transporter family, mfp subunit	2642131-2643306	1175	4120828-4121997	1169	76.1
PA2390	pvdT	(ABC) transporter	2643306-2645297	1991	4121999-4123969	1970	80.6
PA2391	opmQ	hypothetical protein	2645303-2646727	1424	4123962-4125365	1403	70.6
PA2392	pvdP	pyoverdinee biosynthesis protein PvdP	c2648410-2646776	1634	2649784-2651403	1619	70.0
PA2393	pvdM	dipeptidase	2648626-2649972	1346	c2648292-2649641	1349	78.6
PA2394	pvdN	pyoverdinee biosynthesis protein PvdN	2649995-2651275	1280	c2647002-2648273	1271	69.7
PA2395	pvdO	pyoverdinee biosynthesis protein PvdO	2651307-2652161	854	c2646107-2646967	860	79.1
PA2396	pvdF	pyoverdinee biosynthesis protein PvdF	c2653057-2652230	827	2645229-2646041	812	75.7

PA2397	pvdE	the ABC transporter involved in the transport of the pyoverdinee precursor from the cytoplasm into the periplasm	2653435-2655081	1646	c2643358-2645010	1652	76.8
PA2398	fpvA	ferripyoverdine receptor	2655187-2657634	2447	c2640832-2643204	2372	70.3
PA2399	pvdD	pyoverdinee biosynthesis protein PvdD	c2665144-2657798	7346	2627194-2640693	13499	64.1
PA2400	pvdJ	pyoverdinee biosynthesis protein PvdJ	c2671629-2665156	6473	2619339-2627120	7781	55.1
PA2402	pvdI	peptide synthase	c2687178-2671729	15449	2608401-2619335	10934	53.4
PA2413	pvdH	diaminobutyrate-2-oxoglutarate aminotransferase	c2696251-2694842	1409	c4515248-4516720	1472	79.3
PA2424	pvdL	pyoverdinee biosynthesis protein PvdL	c2720694-2707666	13028	c4526235-4539113	12878	76.5
PA2425	pvdG	pyoverdine biosynthesis pro tein PvdG	c2721531-2720767	764	c4539167-4539919	752	66.0
PA2426	pvdS	extracytoplasmic-function sigma-70 factor	c2722175-2722738	563	4540284-4540760	476	84.1

Note: c in position indicates complementary chain.

Supplementary Table 3 | The content of iron-binding siderophore of supernatant of *Pseudomonas* sp. 1502IPR-01 and fraction obtained by XAD-4

batch	iron-binding ability in supernatant (μM equivalents of DFOB in 10 L)	iron-binding ability in fraction obtained by XAD-4 (μM equivalents of DFOB)	proportion of iron-binding ability obtained by XAD-4 to those of supernatant (%)
1	2548.54	2263.41	88.81
2	2483.13	2283.61	91.96
3	2681.74	2434.12	90.77

Note: The method for isolating iron-binding agents with XAD-4 is explained in the method section 'Pyoverdine Production and Purification'. The XAD-4 fraction of our samples contained only one siderophore and this siderophore is pyoverdine (Fig. 4f and Supplementary Fig. 11). Because the XAD-4 fraction covered 88.8~92.0% of the total iron-binding ability of the supernatant, pyoverdine in the XAD-4 fraction is considered the primary iron-binding agent of *Pseudomonas* sp. 1502IPR-01. DFOB indicates Desferrioxamine B

Supplementary Table 4 | ^{1}H NMR data (δ [ppm]) of pyoverdine of *Pseudomonas* sp. 1502IPR-01 (pH 4.3; 30 °C; H_2O/D_2O, 9:1)[a].

KgI	2′	3′							
	2.82	2.34/2.68							
Chr	1	2	3	4NH$^+$	6	7	10	5-NH	
	5.69	2.44/2.71	3.35/3.66	ND	7.85	7.17	7.02	ND	
Amino acids	α-NH	α	Β	γ	δ	ε	ε-NH_2	CHO_Z	CHO_E
Lys^1	8.09	4.26	1.68	1.11/1.22	1.42	3.18	7.74		
Lys^2	8.46	4.07	1.89	1.45	1.68	2.99	ND		
Ser^1	9.19	4.53	3.94						
Ser^2	8.57	4.45	3.84						
Ser^3	8.31	4.45	3.83						
Ser^4	8.11	4.27	3.94						
Ser^5	8.18	4.21	3.94						
FoOHOrn^1	8.19	4.24	1.50/1.64	1.48	3.36/3.44			7.96	8.23
FoOHOrn^2	8.53	4.26	1.79	1.75	3.57/3.61			7.96	8.3

Note: [a] Based on COSY, NOESY, HSQC, HMBC correlations. ND - not detectable.

Supplementary Table 5 | ^{13}C NMR data (δ [ppm]) of pyoverdine of *Pseudomonas* sp. 1502IPR-01 (pH 4.3; 30 °C; H_2O/D_2O, 9:1)[a].

KgI	1′CO	2′ CH_2	3′ CH_2	4′CO	COOH			
	175.4	29	32.6	ND	179.3			
Chr	CO	1	2	3	4a	5	6	
	171.3	57.4	22	35.4	ND	119.7	144	
	6a	7	8	9	10	10a		
	116.9	114.9	144	152.6	100.6	132.8		
Amino acids	CO	α	β	γ	δ	ε	CHO_Z	CHO_E
Lys[1]	173.3	53.8	30.9	21.6	27.6	39.1		
Lys[2]	173.6	54.9	29	22.6	26.5	39.6		
Ser[1]	172.1	56.4	61.2					
Ser[2]	171.8	55.8	61.4					
Ser[3]	172	55.7	61.4					
Ser[4]	172	55.5	61.1					
Ser[5]	171.9	56.4	60.4					
FoOHOrn[1]	174.1	53.8	27.2	22	49.8_E/ 49.9_Z		159.5	163.8
FoOHOrn[2]	174.1	53.9	27.6	22.5	49.8_E/ 49.9_Z		159.6	163.8

Note: [a] Based on COSY, NOESY, HSQC, HMBC correlations. ND - not detectable.

Supplementary Table 6 | Properties of soil used in pot experiments

Soil properties	Value
pH (in water)	8.3
total N	0.45 g kg^{-1}
NO_3-N	8.82 mg kg^{-1}
NH_4-N	1.16 mg kg^{-1}
organic carbon	12.54 g kg^{-1}
$CaCO_3$	10.11%
available P (Olsen-P)	3.82 mg kg^{-1}
available K (NH_4OAc-K)	38.7 mg kg^{-1}
available Fe	1.94 mg kg^{-1}
available Mn	2.21 mg kg^{-1}
available Zn	0.62 mg kg^{-1}
available Cu	2.20 mg kg^{-1}

Supplementary Table 7 | Information on the representative *Pseudomonas* spp. used for the phylogenetic tree analysis

IMG Genome ID/<br>NCBI accession number	Strain name	Type Strain?
2639762715	*P*. extremorientalis BS2774	type strain
GCF_900625005.1	*P*. extremorientalis DSM 15824T	
GCF_001870465.1	*P*. extremorientalis LMG 19695	
GCF_008801565.1	*P*. extremorientalis CCUG 51517	
2663762772	*P*. simiae DSM 18861	type strain
GCF_000934565.1	*P*. simiae PCL1751	
GCF_016134935.2	*P*. simiae K-Hf-L9	
GCF_000963495.1	*P*. simiae PICF7	
2636416017	*P*. azotoformans LMG 21611	type strain
GCF_002091515.1	*P*. azotoformans NBRC 12693	
2639762588	*P*. palleroniana BS3265	type strain
2802429419	*P*. palleroniana MAB3	
2616644927	*P*. lurida LMG 21995	type strain
2802429421	*P*. lurida MYb11	
GCA_014268225.1	*P*. lurida PGSB 3962	
GCA_000801835.1	*P*. lurida AU10973	
2636416169	*P*. libanensis BS2975	
2700989493	*P*. libanensis DSM 17149	type strain
2648501301	*P*. libanensis RIT-PI-g	
GCA_003952245.1	*P*. libanensis DMSP-1	
2636415941	*P*. aeruginosa DSM 50071	
GCF_000006765.1	*P*. aeruginosa PAO1	type strain
GCF_001063005.1	*P*. aeruginosa 231 PPRO	

References

1. Sultana, R. *et al.* A pyoverdin from *Pseudomonas* sp. CFML 95-275. *Z. Naturforsch. C.* **55**, 857–865 (2000).

2. Nicolafrancesco, C. *et al.* Gallium- and iron-pyoverdine coordination compounds investigated by X-ray photoelectron spectroscopy and X-ray absorption spectroscopy. *Inorg. Chem.* **58**, 4935–4944 (2019).
