# Supplementary material for: Microbiome convergence enables siderophore-secreting-rhizobacteria to improve iron nutrition and yield of peanut intercropped with maize
Source: Nat Commun. 2024 Jan 29;15:839. doi: 10.1038/s41467-024-45207-0 (PMC10825131; doi:10.1038/s41467-024-45207-0)
Supplement: Supplementary file 2 — Reporting Summary [file 41467_2024_45207_MOESM2_ESM.pdf]

nature portfolio

Corresponding author(s): Ming Wang, Zhong Wei and Yuanmei Zuo

Last updated by author(s): Sep 17, 2023

Reporting Summary

Nature Portfolio wishes to improve the reproducibility of the work that we publish. This form provides structure for consistency and transparency in reporting. For further information on Nature Portfolio policies, see our Editorial Policies and the Editorial Policy Checklist.

Statistics

For all statistical analyses, confirm that the following items are present in the figure legend, table legend, main text, or Methods section.

n/a	Confirmed	
☐	☒	The exact sample size (*n*) for each experimental group/condition, given as a discrete number and unit of measurement
☐	☒	A statement on whether measurements were taken from distinct samples or whether the same sample was measured repeatedly
☐	☒	The statistical test(s) used AND whether they are one- or two-sided<br>*Only common tests should be described solely by name; describe more complex techniques in the Methods section.*
☒	☐	A description of all covariates tested
☐	☒	A description of any assumptions or corrections, such as tests of normality and adjustment for multiple comparisons
☐	☒	A full description of the statistical parameters including central tendency (e.g. means) or other basic estimates (e.g. regression coefficient) AND variation (e.g. standard deviation) or associated estimates of uncertainty (e.g. confidence intervals)
☐	☒	For null hypothesis testing, the test statistic (e.g. *F*, *t*, *r*) with confidence intervals, effect sizes, degrees of freedom and *P* value noted<br>*Give P values as exact values whenever suitable.*
☒	☐	For Bayesian analysis, information on the choice of priors and Markov chain Monte Carlo settings
☐	☒	For hierarchical and complex designs, identification of the appropriate level for tests and full reporting of outcomes
☐	☒	Estimates of effect sizes (e.g. Cohen's *d*, Pearson's *r*), indicating how they were calculated

Our web collection on statistics for biologists contains articles on many of the points above.

Software and code

Policy information about availability of computer code

Data collection	No software was used for data collection
Data analysis	Most of the analysis were carried out using R version 4.2.2 using standard functions implemented in the base package; Adobe Illustrator version CC, 2020 was used to creating figures; SnapGene version 6.0.2, TBLASTN+ version 2.10.0, MAFFT version 7.427, IQ-TREE2 version 2.1.4-beta and QIIME2 version 2021.8 were used to sequence analysis. Additional details are described in the Methods section and codes for analysis have been deposited to Github repository with the following digital identifier: https://github.com/wnq13579/Peanut-maize-intercropping-microbiome.

For manuscripts utilizing custom algorithms or software that are central to the research but not yet described in published literature, software must be made available to editors and reviewers. We strongly encourage code deposition in a community repository (e.g. GitHub). See the Nature Portfolio guidelines for submitting code & software for further information.

Data

Policy information about availability of data

All manuscripts must include a data availability statement. This statement should provide the following information, where applicable:

- Accession codes, unique identifiers, or web links for publicly available datasets
- A description of any restrictions on data availability
- For clinical datasets or third party data, please ensure that the statement adheres to our policy

We deposited the following sequences at NCBI under the listed accession numbers: Raw data of 16S rRNA amplicon sequencing used in this study are deposited at

the NCBI database under BioProject PRJNA788265 (https://dataview.ncbi.nlm.nih.gov/object/PRJNA788265). 16S rRNA gene sequences of 46 isolated rhizobacteria are deposited on GenBank with accession OL824679 (https://www.ncbi.nlm.nih.gov/nuccore/OL824679) - OL824724 (https://www.ncbi.nlm.nih.gov/nuccore/OL824724). The Pseudomonas sp. 1502IPR-01 reference strain genome sequence, determined for this study, is deposited at the NCBI database under BioProject PRJNA788188 (https://dataview.ncbi.nlm.nih.gov/object/PRJNA788188). The raw data generated in this study that support the findings of this study are available in the Figshare repository under accession cod 10.6084/m9.figshare.24648306 (https://doi.org/10.6084/m9.figshare.24648306).

Research involving human participants, their data, or biological material

Policy information about studies with human participants or human data. See also policy information about sex, gender (identity/presentation), and sexual orientation and race, ethnicity and racism.

Reporting on sex and gender	Human research participants are not involved in this study.
Reporting on race, ethnicity, or other socially relevant groupings	Human research participants are not involved in this study.
Population characteristics	Human research participants are not involved in this study.
Recruitment	Human research participants are not involved in this study.
Ethics oversight	Human research participants are not involved in this study.

Note that full information on the approval of the study protocol must also be provided in the manuscript.

Field-specific reporting

Please select the one below that is the best fit for your research. If you are not sure, read the appropriate sections before making your selection.

☐ Life sciences ☐ Behavioural & social sciences ☒ Ecological, evolutionary & environmental sciences

For a reference copy of the document with all sections, see nature.com/documents/nr-reporting-summary-flat.pdf

Ecological, evolutionary & environmental sciences study design

All studies must disclose on these points even when the disclosure is negative.

Study description	We used 48 rhizosphere soil samples and associated plant samples across maize/peanut intercropping, monocropping peanut and monocropping maize to determine the relationship between plant-soil iron level and rhizosphere bacterial communities affected by intercropping. We screened out a representative isolate Pseudomonas sp. 1502IPR-01, from 46 bacterial isolates with high siderophore-secreting ability from intercropping rhizosphere, and its siderophore pyoverdine, and revealed that peanut iron nutrition and yield are enhanced by iron-chelating pyoverdine. Collectively, we demonstrate that the convergence of siderophore-secreting-rhizobacteria plays a pivotal role in improving the performance of iron-limited peanut plants through a complex plant-microbe-plant interaction.
Research sample	1.Soil and associated DNA from 48 rhizosphere samples and associated plant samples of maize/peanut intercropping, monocropping peanut and monocropping maize.<br>2.46 bacterial isolates with high siderophore-secreting ability from 12 intercropping peanut rhizosphere samples.<br>3.One Pseudomonas isolate with the high siderophore-secreting ability and its siderophore-pyoverdine.<br>4.Soil from 48 rhizosphere samples and associated plant samples of maize/peanut intercropping and monocropping peanut grown in normal and sterilized soil treated by Pseudomonas sp. 1502IPR-01 and its siderophore pyoverdine.<br>5.Soil from 56 rhizosphere soil samples and associated plant samples of monocropping peanut from two fields site treated by Pseudomonas sp. 1502IPR-01 and its siderophore pyoverdine.<br>6.Soil from 16 rhizosphere samples and associated plants samples of monocropping peanut treated by siderophore null mutant.
Sampling strategy	1. 48 rhizosphere soil samples and associated plant samples were collected from individual plants of maize/peanut intercropping, monocropping peanut and monocropping maize grown in iron-limited calcareous soil in pot at four time points associated with peanut iron level. The excess soil was first gently shaken from the roots and the remaining soil attached to the roots was considered as the rhizosphere soil. Before collecting rhizosphere samples, the roots of intercropping maize and intercropping peanut were completely separated through different root colour. We used Illumina sequencing to reveal the whole bacterial community structure for each plant in a cultivation-independent way, analyzed the iron level of soil and associated plant samples and isolated 46 bacterial isolates with the high siderophore-secreting ability through CAS test.<br>2.Siderophore of Pseudomonas sp. 1502IPR-01 was purified by XAD-4 polymeric adsorbent resin and semi-preparative HPLC and its structure was identified by combination of LC-HRMS, Orbitrap MS and NMR.<br>3.The rhizosphere soil samples and associated plant samples of greenhouse and field experiments with Pseudomonas spp. strains, siderophore pyoverdine and mutant were collected according to the similar sampling strategy described above.
Data collection	N.-Q. W., T.-Q. W. Q.-F. L., K.-G. W., Z.-C. D., Z.-G. C., W.-Q., J.-D., L.-N. and J.-Y. C. performed and collected the data. (see author contributions for detail).<br>SPAD values were measured using a chlorophyll meter (Konica-Minolta, Osaka, Japan).<br>The absorbance of the solution were measured using an ultraviolet-visible spectrophotometer 2600i (Shimadzu, Kyoto, Japan).

	The iron concentration was measured by inductively coupled plasma-optical emission spectrometry (ICP-OES) using a 7300DV system (Perkin Elmer, Waltham, USA).<br>Biomass was measured using a electronic scale (Sartorius, Gottingen, Germany).<br>Purified amplicons were pooled in equimolar and paired-end sequenced on an Illumina MiSeq PE300 platform (Illumina, San Diego, USA). For Illumina sequencing, the prepared libraries were then used for paired-end Illumina sequencing (2 × 150 bp) on an Illumina HiSeq X Ten machine (Illumina, San Diego, USA). For Pacific Biosciences sequencing, a ~10kb insert library was prepared and sequenced on one SMRT cell using standard methods. Mass spectrometry: LC-HRMS spectra were obtained on an Agilent 6530 Q-TOF mass spectrometer coupled to an Agilent 1260 HPLC (Agilent Technologies GmbH, Waldbronn, Germany); Thermo Orbitrap MS (Orbitrap Fusion Lumos, Thermo Fisher Scientific, San Jose, USA); ESI-QQQ-MSMS (Xevo TQ-S micro, Waters, Milford, USA).<br>NMR data were obtained using (operating at 500.13 MHz for 1H NMR and 125.75 MHz for 13C NMR) (Bruker Biospin GmbH, Karlsruhe, Germany). All spectra were measured at 303 K. The residual solvent signals were used for referencing spectra in the 1H and 13C dimensions.<br>UV/Vis: The UV spectra were recorded by a SPD-M20A Shimadzu photodiode array detector (PDA, detection 190 - 800 nm) (Shimadzu, Kyoto, Japan) connected to a Shimadzu HPLC system (LC-20AR, Shimadzu, Japan)
Timing and spatial scale	Greenhouse experiments for maize/peanut intercropping, monocropping peanut, monocropping maize were carried out between May and August, 2015 at 46, 53, 63 days post sowing (dps). Siderophore of Pseudomonas sp. 1502IPR-01 purification and its structure identification were carried out between October, 2019 and July, 2021. Greenhouse and field experiments with Pseudomonas sp. 1502IPR-01 and its siderophore pyoverdine were carried out between May and October, 2020 and application were performed at anthesis (50 dps), pod bearing (64 dps), and fruit-swelling stage (75 dps) . Greenhouse experiments with siderophore null mutant were carried out between May and August, 2022 and application were performed at anthesis (50 dps), pod bearing (64 dps), and fruit-swelling stage (75 dps). Field experiments were carried out in different sites separated by about 500 km between May and October, 2020. Field application were carried out. At anthesis (53 dps in Beijing and Puyang), pod bearing (65 dps in Beijing and 68 dps in Puyang), and fruit-swelling (79 dps in Beijing and 84 dps in Puyang)
Data exclusions	No data exclusion
Reproducibility	All measurements were carried out in at least triplicate. The field experimental sampling was repeated across two independent field sites separated by about 500 km to ensure replication.
Randomization	The sampling was randomized to ensure a balanced sample representation across sites and plants. Samples were randomized during lab experiments, plants were randomized during greenhouse and field experiments (see methods for detail).
Blinding	We used abstract labels for the different bacterial isolates during the lab and greenhouse and field experiments.

Did the study involve field work? ☒ Yes ☐ No

Field work, collection and transport

Field conditions	Agricultural fields in Beijing and Puyang, China. In Beijing, the average temperature from May to September was 25.6℃, and the annual precipitation was 603 mm. The type of soil in Beijing is calcic cambisol. The soil property was pH 7.9, total N 0.038%, available P<br>(Olsen-P) 5.12 mg·kg-1, available K (NH4OAc-K) 60.32 mg/kg, available Fe 3.35 mg/kg. In Puyang, The average temperature from May to September was 25.4℃, and the annual precipitation was 604.1 mm. The type of soil in Henan is fluvo-aquic soil. The soil property was pH 7.7, total N 0.041%, available P (Olsen-P) 7.42 mg/kg, available K (NH4OAc-K) 70.2 mg/kg, available Fe 3.74 mg/kg.
Location	Beijing (116°10′ E, 39°39′ N) and Puyang, Henan(115°9′ E, 36°7′ N)
Access & import/export	The sample collection did not involve sensitive ecosystems. Minimal amounts of soil were collected at each sites (a few grams per sample)
Disturbance	No disturbance caused by this work.

Reporting for specific materials, systems and methods

We require information from authors about some types of materials, experimental systems and methods used in many studies. Here, indicate whether each material, system or method listed is relevant to your study. If you are not sure if a list item applies to your research, read the appropriate section before selecting a response.

Materials & experimental systems

n/a	Involved in the study
☒	☐ Antibodies
☒	☐ Eukaryotic cell lines
☒	☐ Palaeontology and archaeology
☒	☐ Animals and other organisms
☒	☐ Clinical data
☒	☐ Dual use research of concern
☐	☒ Plants

Methods

n/a	Involved in the study
☒	☐ ChIP-seq
☒	☐ Flow cytometry
☒	☐ MRI-based neuroimaging

Dual use research of concern

Policy information about dual use research of concern

Hazards

Could the accidental, deliberate or reckless misuse of agents or technologies generated in the work, or the application of information presented in the manuscript, pose a threat to:

No	Yes
☒	☐ Public health
☒	☐ National security
☒	☐ Crops and/or livestock
☒	☐ Ecosystems
☒	☐ Any other significant area

Experiments of concern

Does the work involve any of these experiments of concern:

No	Yes
☒	☐ Demonstrate how to render a vaccine ineffective
☒	☐ Confer resistance to therapeutically useful antibiotics or antiviral agents
☒	☐ Enhance the virulence of a pathogen or render a nonpathogen virulent
☒	☐ Increase transmissibility of a pathogen
☒	☐ Alter the host range of a pathogen
☒	☐ Enable evasion of diagnostic/detection modalities
☒	☐ Enable the weaponization of a biological agent or toxin
☒	☐ Any other potentially harmful combination of experiments and agents
