# Supplementary material for: Microbiome convergence enables siderophore-secreting-rhizobacteria to improve iron nutrition and yield of peanut intercropped with maize
Source: Nat Commun. 2024 Jan 29;15:839. doi: 10.1038/s41467-024-45207-0 (PMC10825131; doi:10.1038/s41467-024-45207-0)
Supplement: Supplementary file 17 — Description of Additional Supplementary Files [file 41467_2024_45207_MOESM17_ESM.pdf]

1. File Name: Supplementary Data 1

Description: Correlation and linear regression metrics of relative abundance of genus that differentiated between intercropping peanut and monocropping peanut with plant-soil iron nutrition of peanut.

2. File Name: Supplementary Data 2

Description: ASV feature table generated by QIIME2.

3. File Name: Supplementary Data 3

Description: Sample information of 16S sample.

4. File Name: Supplementary Data 4

Description: Taxonomy table generated by QIIME2.

5. File Name: Supplementary Data 5

Description: Representative sequences generated by QIIME2.

6. File Name: Supplementary Data 6

Description: Rooted tree generated by QIIME2.

7. File Name: Supplementary Data 7

Description: Phylogenetic tree of *Pseudomonas* sp. 1502IPR-01 and other strians of *Pseudomonas*.

8. File Name: Supplementary Data 8

Description: Phylogenetic tree of siderophore-secreting rhizobacteria in the study.

9. File Name: Supplementary Data 9

Description: Structure of pyoverdine of Pseudomonas sp. 1502IPR-01 (ChemDraw file).

10. File Name: Supplementary Data 10

Description: Proposed structure-fragmentation pathways of the chromophore of pyoverdine in supplementary Fig. 11 (ChemDraw file).

11. File Name: Supplementary Data 11

Description: Ions observed after collision activation of $[M + H]^+$ and $[M – H_2O – CO_2$

+ H]$^+$ ions of pyoverdine. in supplementary Fig. 12-14 (ChemDraw file).

12. File Name: Supplementary Data 12

Description: Key ^{1}H–^{1}H COSY (black thick lines) and key HMBC correlations (black arrows) of pyoverdine. in supplementary Fig. 21 (ChemDraw file).

13. File Name: Supplementary Data 13

Description: Raw data for calculating the correlation and linear regression metrics of relative abundance of genus with plant-soil iron nutrition of peanut shown in Supplementary Data 1, containing two parts of data: 1) the active iron of young leaves and available iron of peanut in intercropping peanut and monocropping peanut pot experiment, which have been offered in "fig 3b" sheet in Source data file; 2) the relative abundance of each genus in peanut rhizosphere, which could be calculated according to Supplementary Data 2, 3 and 4.
